# Expression and regulation of phenol-soluble modulins and enterotoxins in foodborne *Staphylococcus aureus*

**DOI:** 10.1186/s13568-018-0717-x

**Published:** 2018-11-22

**Authors:** Xiaoxiao Wu, Miao Yang, Xin Fang, Shiqi Zhen, Jie Zhang, Xiangying Yang, Ling Qiao, Yang Yang, Chi Zhang

**Affiliations:** 1 National Center of Supervision Inspection on Processed Food & Food Additives Quality, Nanjing Institute of Product Quality Inspection, No. 3 Jialingjiang East Street, Nanjing, 210019 China; 2 Key Laboratory of Child Development and Learning Science (Ministry of Education), School of Biological Science & Medical Engineering, Southeast University, No. 2 Sipailou Street, Nanjing, 210018 China; 3 Jiangsu Provincial Center for Disease Control and Prevention, No. 172 Jiangsu Street, Nanjing, 210009 China; 4 Beijing Entry-Exit Inspection and Quarantine Bureau Inspection and Quarantine Technical Center, No. 6 Tianshuiyuan Street, Chaoyang District, Beijing, 100026 China

**Keywords:** *Staphylococcus aureus*, Phenol-soluble modulin, Staphylococcal enterotoxin, Foodborne, Co-expression

## Abstract

Although high levels of staphylococcal phenol-soluble modulins (PSMs) in clinical methicillin-resistant *Staphylococcus aureus* (MRSA) has been shown to correlate with bacterial virulence, the PSMs expression in foodborne *Staphylococcus aureus* (*S. aureus*), as well as its association with staphylococcal food poisoning (SFP) was not yet clear. We collected a panel of 350 foodborne and 127 clinic-derived *S. aureus* strains and compared their PSMs expression. Overall, foodborne strains exhibited higher PSMs than clinical isolates, indicating a potential pathological significance of PSMs in staphylococcal food contamination. Furthermore, PSMs expression and staphylococcal enterotoxins (SEs) levels in relation to antibiotic sensitive and resistant strains were analysed. While the co-expression of PSMs and SEs was confirmed, one typical foodborne strain simultaneously yielding PSMs, SEB and SED was selected. By comparing this wildtype strain to a series of gene-deficient mutants, we concluded that PSMs and SEs expressions both relied on staphylococcal accessory regulator A initiation in the early stage of accessory gene regulator control, yet their succedent regulations differentiated to RNAIII-dependent and independent, respectively. These data provided preliminary insight into PSMs and SEs expression in foodborne *S. aureus*, and may guide the further studies on PSMs effects in SFP.

**Electronic supplementary material:**

The online version of this article (10.1186/s13568-018-0717-x) contains supplementary material, which is available to authorized users.

## Introduction

*Staphylococcus aureus* (*S. aureus*) is not only a common cause of clinical infection, but also one of the leading foodborne pathogen worldwide (Kadariya et al. 2014). During recent decades, persistent antibiotic exposure has produced various resistant strains, among which Methicillin-resistant *S. aureus* (MRSA) is among the most notorious. In the 1990s, the emergence of Community-associated MRSA (CA-MRSA) indicated that MRSA has spread out of the hospital and threatened the public health (Argudín and Mendoza 2010). The morbid *S. aureus* strains, multidrug-resistant variants in particular, are versatile in causing diseases ranging from mild toxic reactions to fatal infections such as pyemia, endocarditis and pneumonia (Klevens et al. 2007; Otto 2012; Waters et al. 2011).

As a recently discovered staphylococcal cytolysin family, phenol-soluble modulins (PSMs) are a group of small amphipathic peptides with α-helical structure. In *S. aureus*, at least 7 PSMs have been discovered: PSMα1 to PSMα4, PSMβ1 and PSMβ2, and the *S. aureus* δ-toxin (Chatterjee and Otto 2013; Otto 2014; Peschel and Otto 2013; Wang et al. 2007). *Psm* genes, which are located on the core genome, generate the shorter (20–25 amino acids) α-type and the longer (44 amino acids) β-type peptides through post-translational cleavage (Wang et al. 2007). Among the typical strains separated from clinic, PSMs production is more abundant in CA-MRSA than in Hospital-associated MRSA (HA-MRSA), and extremely high in high-virulence strains such as USA300 and USA400 (Otto 2014; Peschel and Otto 2013). Generally, the physiological effects of PSMs include host cell lysing, biofilm forming, pro-inflammation and antimicrobial effect (Chatterjee and Otto 2013; Peschel and Otto 2013; Wang et al. 2007; Cheung et al. 2012; Geiger et al. 2012; Joo et al. 2011; Somerville et al. 2003; Surewaard et al. 2012, 2013; Tsompanidou et al. 2013). These multifaceted properties indicate that PSMs represent a novel global virulence factor, and also a kind of essential molecule in organism colonization during the commensal lifestyle (Otto 2014; Peschel and Otto 2013).

In the process of staphylococcal food poisoning (SFP), the key toxins are staphylococcal enterotoxins (SEs), a family of thermostable and gastrointestinal protease-tolerant superantigen (Marrack and Kappler 1990). The SEs vary greatly among *S. aureus* strains, which are regulated by multiple and often overlapping pathways under the influence of environmental factors (Fisher et al. 2018). Once the food is contaminated with *S. aureus*, the organism will be allowed to grow to a high cell density under appropriate environment, triggering the production of SEs. After the ingestion, victims develop abdominal pain, intense diarrhea and vomiting (Marrack and Kappler 1990). Since 2002, CA-MRSA strains have been successively separated from SFP outbreaks, indicating that CA-MRSA is strongly correlated with SFP in addition to clinical infection (Jones et al. 2002; Zhang et al. 2013).

Since PSMs and most of SEs are controlled by Agr system (Zhang et al. 2013; Carnes et al. 2010; Boisset et al. 2007), their simultaneous production in the late-exponential phase infers a possible synergistic effect. Moreover, the surfactant-like properties of PSMs may accelerate microbial spread in food matrix (Tsompanidou et al. 2013), and the antimicrobial effect may benefit the colonization and multiplying (Joo et al. 2011). According to an “outside-in” signaling mechanism, staphylococcal superantigens progressively interact with host cells to stimulate the immune reaction, accompanied by cytolysins which facilitate the disease production (Stach et al. 2014). To date, there has been no report on PSMs expression in foodborne *S. aureus* and its relation to antibiotic resistance. Furthermore, the co-expression and regulation of PSMs and SEs in foodborne strains have not yet been verified. Here, in order to initiate a research area into PSMs effect in food safety, we collected a panel of *S. aureus* isolates from various food, and assessed their PSMs production by a high-resolution mass spectrometry. Then, the correlation between PSMs and SEs expression, as well as the possible regulation pathways, were assayed in wild type and gene-deficient strains.

## Materials and methods

### *S. aureus* strains

A panel of foodborne *S. aureus* isolates was collected by the laboratories of the National Center of Food Testing & Supervision and Jiangsu Provincial Center for Disease Control and Prevention. In addition, a number of *S. aureus* strains were derived from local hospital as the control. Twenty standard *S. aureus* strains, including USA300 and USA400, were also included (Additional file 1: Table S1). All strains were spread on Brad-parker Agar (Luqiao, China), and then confirmed to be plasma-coagulase positive. Morphologically typical clones were isolated and incubated in Tryptic Soybean broth (Luqiao, China) at 37 °C for subsequent studies.

### Antibiotic resistance assay

Susceptibilities of *S. aureus* stains were tested using the Sensititre Susceptibility Plates for Clinical Non-Fastidious Organism PRCM1F (ThermoFisher, USA). One 96-well plate contained 7 serially diluted antibiotics coated on the wells: Oxacillin, Erythromycin, Tetracycline, Clindamycin, Ciprofloxacin, Vancomycin and Chloramphenicol. The assay was conducted according to the manufacturer’s instructions. MIC values were determined by the terminal well of precipitation growth, and the susceptibilities were determined in accordance with NCCLS criteria.

### Detection of PSMs expression

After overnight growth, *S. aureus* cells were separated by centrifugation. The culture supernatants were incubated at a 1:1 ratio with 1-butanol at 37 °C for 2 h. The mixture was then centrifuged and the layer containing 1-butanol was collected and dried. Ultrapure water was used to redissolve the dried sample, which was immediately filtered through a syringe filter before LC–MS analysis.

A high performance liquid chromatography–quadrupole time-of-flight mass spectrometry (HPLC–QTOF) system (Agilent, USA) was used to analyze PSMs levels in the treated supernatant qualitatively and quantitatively. The chromatographic separation was performed with a Zorbax 300 Å SB-C8 column (3.5 µm, 2.1 × 150 mm) in series with a flow rate of 0.5 mL/min and a binary solvent system with mobile phase A (0.1% formic acid in water) and mobile phase B (0.1% formic acid in acetonitrile). The ESI experiments were carried out by Dual AJS ESI ion source. Signals acquired in MS mode were used to make a preliminary identification, and data obtained in targeted MS/MS mode were searched in the online Mascot database (http://www.matrixscience.com) to confirm the peptide sequence. As standard substances, all PSM peptides were synthesized by commercial vendors at > 95% purity with an N-terminal *N*-formyl methionine modification, as found in naturally occurring PSMs.

### SEs levels

The presence of SEs in the *S. aureus* culture supernatants was firstly detected using the mini-Vidas Staphylococcal Enterotoxin II Kit (Bio Mérieux, France). SE-positive strains were selected and the expressions of SEA to SEE were quantified using *S. aureus* Enterotoxin ABCDE ELISA Kit (Ridascreen, Germany). Based on the ELISA principle, the concentrations of each SEs were determined by the standard curves derived from a series of standard samples.

### Construction of gene-deficient strains

Different mutants derived from a typical foodborne *S. aureus* strain were constructed, in which the whole *agr* gene, *RNAIII* gene, *agrA* gene, *sarA* gene and *rot* gene were deleted, respectively. First, the upstream and downstream fragments (400 bp each) of each target gene were amplified, and then the recombinant genes in which the upstream and downstream fragments flanked the erythromycin (Em) resistance gene were constructed. The recombinant genes were inserted into the shuttle vector pBT2 for allele replacement. The plasmids and primers used in this study are shown in Additional file 1: Table S2.

The *S. aureus* strain RN4220 at early-exponential phase was suspended in 0.5 M sucrose, and kept on ice to yield electro-competent cells. The cells were transferred to a Gene Pulser cuvette, and the electro-transfer was performed on an ECM830 electroporator (BTX, USA). The electric settings were as follows: Voltage, 2.5 kV; capacitor, 50 μF; resistance, 200 Ω. After electroporation, the cells were immediately transferred into 400 μL of TSB for 1 h, placed on Em-containing medium and incubated overnight. Subsequently, the plasmid was extracted and electro-transformed into the target strain using the same procedure. The clones were incubated in B-medium at 30 °C for 12 h and left in 40 °C to grow overnight. The 1:100 diluted culture was inoculated into fresh B-medium, and 1 μL of overnight culture was spread onto the agar plate until Em-resistant, Chloramphenicol (Cm)-sensitive clones were found. The mutations were confirmed by PCR amplification with specific primers.

### Statistical analysis

The PSMs levels between *S. aureus* groups were compared using the Student’s t test and One-way ANOVA. Linear analysis was applied to determine the correlation between PSMs and SEs co-expression. All statistics were performed using InStat 3.06 Software (Graphpad, La Jolla, CA, USA).

### Accession numbers

All sequences of agr, RNAIII, agrA, sarA and rot were retrieved from the whole genome sequence of *S. aureus* strain USA300 (Accession No. AASB02000001.1).

## Results

### PSMs expression in foodborne *S. aureus*

**Fig. 1:**
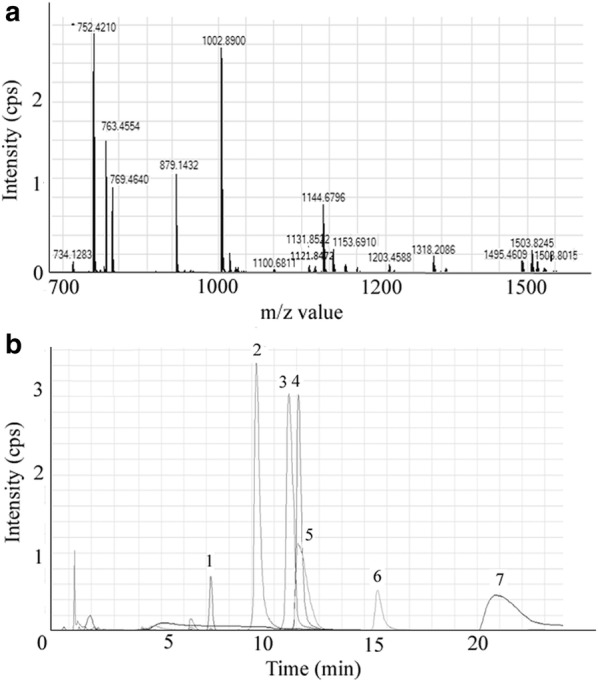
QTOF MS spectrum and ion chromatograms of PSMs. **a** QTOF MS spectrum of PSMs in the mixed standard solution. **b** Extracted ion chromatograms of PSMs in USA 300 culture supernatant: 1, PSMβ2 (m/z 1495.4609); 2, PSMα3 (m/z 879.1432); 3, PSMα2 (m/z 769.4640); 4, PSMα1 (m/z 1144.6796); 5, δ-toxin (m/z 1503.8245); 6, PSMβ1 (m/z 1508.8015); 7, PSMα4 (m/z 734.1283)

**Fig. 2:**
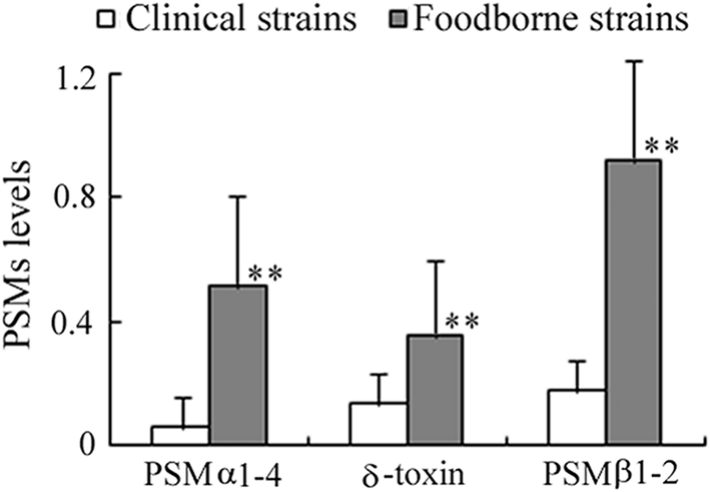
PSMs expression in foodborne and clinically-derived *S. aureus*. The PSMα1-4, δ-toxin and PSMβ1-2 levels in foodborne strains were significantly higher than those in clinical strains, respectively. Results were expressed as the ratio of PSMs levels in tested strains to those in USA300. **p < 0.01

A high resolution HPLC-ESI-QTOF approach was established to assay PSMs expressed by *S. aureus* strains. In positive ionization mode, the [M+nH]^n+^ ion peaks of 7 target PSMs were detected according to their accurate *m/z* ratios, charges and retention time (Table 1). Further, data obtained in MS/MS were substituted into Mascot Software to confirm the peptide. The query results were in accordance with the published sequences and modification. Meanwhile, the representative mass spectrum of 7 PSMs in the supernatant were also identical to those of the synthesized peptides. The QTOF MS spectrum of PSMs in the mixed standard solution, as well as the extract ion chromatography (EIC) of 7 PSMs in USA300 are shown in Fig. 1. Taken the PSMs level of USA300 as 100%, PSMs expressed by 3 different operons (PSMα1-4, δ toxin and PSMβ1-2) in all strains were normalized (Additional file 1: Table S3). As shown in Fig. 2, the average PSMs levels of 350 food-derived strains were significantly higher than those of 127 isolates separated from patients (*p *< 0.01). In addition, among the standard *S. aureus*, only the CA-MRSA strains USA300 and USA400 exhibited extremely high PSMs production. In contrast, PSMs in most of standard HA-MRSA and methicilin susceptible *Staphylococcus aureus* (MSSA) strains were lower than 20% of those in USA300.

**Table 1:** Amino acid sequence, m/z ratios and retention times of *S. aureus* PSMs

PSMs	Amino acid sequence	MS(m/z ratios, Da)^a^		RT (min)
PSMα1	fMGIIAGIIKVIKSLIEQFTGK	763.4554(t)	1144.6842(d)	12.073
PSMα2	fMGIIAGIIKFIKGLIEKFTGK	769.4640(t)	1153.6964(d)	11.639
PSMα3	fMEFVAKLFKFFKDLLGKFLGNN	879.1432(d)	1318.2228(d)	10.207
PSMα4	fMAIVGTIIKIIKAIIDIFAK	734.1283(t)	1100.6982(d)	20.819
PSMδ-toxin	fMAQDIISTIGDLVKWIIDTVNKFTKK	1002.8844(t)	1503.8245(d)	12.056
PSMβ1	fMEGLFNAIKDTVTAAINNDGAKLGTSIVSIVENGVGLLGKLFGF	1131.8610(q)	1508.8015(t)	15.588
PSMβ2	fMTGLAEAIANTVQAAQQHDSVKLGTSIVDIVANGVGLLGKLFGF	1121.8514(q)	1495.4609(t)	8.190

^a^(d), (t) and (q) respectively stand for doubly, triply and quadruply charged ions

### PSMs levels in drug-resistant strains

**Fig. 3:**
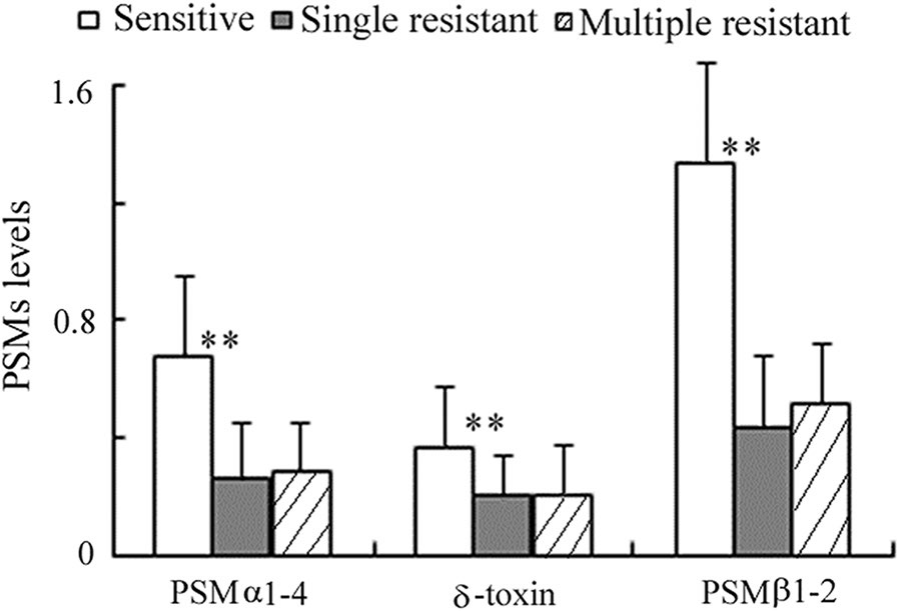
PSMs expression in sensitive and drug-resistant foodborne *S. aureus*. The PSMs in single and multiple antibiotic resistant strains were significantly lower than those in sensitive strains, respectively. Results were expressed as the ratio of PSMs levels in tested strains to those in USA300. **p < 0.01

PSMs expression in common drug-resistant foodborne strains was analyzed. In all food-derived *S. aureus*, 77.4% (271/350) exhibited antibiotic resistance, among which 216 strains were multidrug resistant. The single or multiple antibiotic-resistant strains both exhibited markedly lower PSMs levels than the sensitive counterparts (Fig. 3). In the drug-resistance *S. aureus*, MRSA and vancomycin intermediate *Staphylococcus aureus* (VISA) strains produced mildly higher PSMs compared with other isolates, but these differences did not reach statistical significance (data not shown).

### Co-expression of PSMs and SEs in foodborne *S. aureus*

SEA to SEE production in the culture supernatant of foodborne *S. aureus* was detected. The differences of PSMs levels between SE-positive and negative strains were not significant (data not shown). Yet, high correlations between SEs and PSMs productions were observed in the SE-positive *S. aureus*, indicating co-expression of PSMs and SEs. Table 2 lists the correlation coefficients and statistics between each SEs and PSMs.

**Table 2:** Correlation coefficients between SEs and PSMs levels in foodborne *S. aureus*

Correlation coefficients	SEA	SEB	SEC	SED	SEE
PSMα	0.4065*	0.5344**	0.5430**	0.6006**	0.4120*
δ toxin	0.3552	0.6214**	0.4115*	0.6522**	0.3260
PSMβ	0.5251*	0.5501**	0.5334**	0.5365**	0.4661*

* *p *< 0.05, ** *p *< 0.01

### Regulation of PSMs and SEs co-expression

**Fig. 4:**
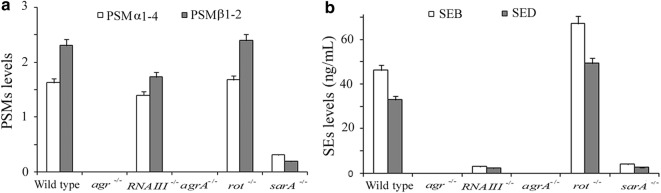
PSMs and SEs expression in representative foodborne *S. aureus* and its variants. Results were Mean ± SD of three experimental replications. **a** PSMs expression, expressed as the ratio of PSMs in tested strains to those in USA300. **b** SEs expression

A representative foodborne *S. aureus* strain, which simultaneously produced PSMs, SEB and SED in high levels, was selected for further study. A series of regulatory gene deficient strains derived from ZJY55 were constructed by homologous recombination, generating *agr*^−/−^, *RNAIII*^−/−^, *agrA*^−/−^, *rot*^−/−^ and *sarA*^−/−^ strains. The expressions of PSMs and SEs in wildtype and the derived strains were detected, and the possible mechanisms of gene regulation were summarized. As shown in Fig. 4, the expressions of PSMs, SEB and SED were diminished in the *agr*^−*/*−^ and *agrA*^−*/*−^ mutants, whereas, the *RNAIII* ^−*/*−^ strain exhibited mildly decreased PSMs but sharply reduced SEs. In the *rot* gene deficient strain, the SEs levels significantly increased while the PSMs levels remained steady. Moreover, PSMs and SEs were both markedly reduced in the *sarA* deficient isolate.

## Discussion

It is generally recognized that highly pathogenic CA-MRSA strains produce higher amount of PSMs than HA-MRSA and MSSA, suggesting a tight correlation between PSMs and virulence (Otto 2014). To date, most studies on PSMs expression were conducted using typical strains separated from patients, including CA-MRSA, HA-MRSA and MSSA (Queck et al. 2009). PSMα links the key roles of cell lysis, and also contributes to biofilm forming (Otto 2014; Peschel and Otto 2013). In addition, PSMs contribute to phenotypes not associated with infection, thereby representing the original mechanisms for bacteria survival, out of which the function of virulence factors evolved (Otto 2014; Periasamy et al. 2012). Food-derived *S. aureus*, usually separated due to the positive reaction of plasma-coagulase, was deemed to show dual capability for food colonization and pathogenicity. During food contamination, a serious of surface proteins, cytolins and superantigens are successively expressed, facilitating the *S. aureus* dissemination and colonization. In the foodborne *S. aureus*, expressions of PSMs and its correlations with drug resistance have not been reported. Here, we collected *S. aureus* strains from various foods to detect the PSMs levels, as well as the pattern of their antibiotic resistance. The foodborne *S. aureus* exhibited relatively higher PSMs expressions in contrast to the clinical isolates, inferring a marked significance of PSMs in the lifecycle of foodborne isolates. Generally, MRSA with multiple antibiotic resistances exhibited relatively high capability in host invasion and existence. In some strains, the emerging of anti-drug resistance was accompanied with the lost of certain virulence genes. When bacteria accepted the plasmid containing resistance genes, the levels of toxic factors might shift (Soto 2009). Here, we revealed that antibiotic-resistant foodborne *S. aureus* synthesized a lower PSMs levels than the sensitive isolates. It indicated that PSMs could be involved in the “original” virulence of *S. aureus* in promoting colonization or emulsifying nutrients (Peschel and Otto 2013), and might be impaired during the acquirement of the antibiotic-resistant properties to adapt the environment.

*S. aureus* secretes a wide variety of virulence factors which are strictly controlled by a complex regulatory network (Kadariya et al. 2014; Delgado et al. 2008). PSMs and most SEs are strictly controlled by Agr quorum sensing, yet their mechanisms are distinct. SEB and SED are both widely distributed superantigens in SFP which are regulated by the ordinary Agr pathway (Novick 2003). In contrast, the Agr control of PSMs is RNAIII-independent. PSMs expression is triggered by direct binding of *agrA* to the *psm* promoter regions, which may coupled with additional regulation other than by *agr* (Queck et al. 2009). In this study, we observed the co-expression between PSMs and SEs in foodborne *S. aureus*. Then, a series of mutants of the typical isolate simultaneously producing PSMs, SEB and SED were used to investigate the regulation of gene expression. The whole Agr system was essential for the high production of both SEs and PSMs, and the co-expression hinges on *sarA* binding onto the *agr* promoter region to initiate transcription (Queck et al. 2008; Zielinska et al. 2011). Further, SEs production was under the regular control of RNAIII via removing *rot* inhibition, whereas, the expression of PSMs was only mildly downgraded by RNAIII, yet uninfluenced in the *rot* gene deleted strain. Accordingly, we concluded that during the early stage of Agr control, PSMs and SEs expressions both rely on the *agrA/B* quorum sensing initiated by *sarA*. However, the subsequent regulations of SEs and PSMs were RNAIII-dependent and independent, respectively. So, additional regulatory elements may be involved in PSMs expression.

An LC–QTOF mass spectrum was employed for PSMs detection in the supernatants of *S. aureus* culture. This high-resolution methodology could accurately discriminate each target peptides from simply pre-treated samples, avoiding the disturbance of molecules with similar *m/z* values. In addition, the output file of ms/ms mode could be searched in the database of protein mass spectrum to confirm the primary structure and modifications. In a previous investigation, according to the imprecise *m/z* detected by HPLC system coupled with an Ion Trap mass spectrometer, the *m/z* 1046.1 peak was initially suggested to be an ADM2 fragment, but subsequently proven to be derived from PSMα after antimicrobial analysis (Joo et al. 2011). Here, the LC–QTOF method confirmed the precise *m/z*, charges, experimental condition and procedures, thus categorically averted the erroneous assessment for target peptides. Furthermore, this approach is preferable to assay peptides in complicated substrates such as food and environmental samples due to its high resolution. We also detected PSMs in artificial contaminated milk and meat using this method, and found massive interfering peptides with similar *m/z* to PSMs, which were impossible be differentiated by former MSD Trap SL mass spectrometer.

Recently, an “outside-in” signaling model has been illustrated by Stach et al. (2014), describing a possible staphylococcal virulence mechanism via the combination of superantigens and cytolins. In this model, the pathogenic superantigen binds to epithelial cells by the aid of a certain cytolysin, and thereby triggers the cascade of preinflammatory signals to disrupt the permeability barrier. Then, the produced cytokines/chemokines attract cells of innate and adaptive immune system to induce massive inflammation and facilitate the disease production (Chatterjee and Otto 2013). However, no combination of superantigen and cytolin has been verified to perform a synergistic reaction in SFP through “outside-in signaling”. The statistical data of PSMs and SEs’ correlation and co-expression raised the possibility that they may act as mutual promotional elements in food poisoning. Further evidence on cell lines and animal models are now indicated to investigate the pathogenic role of PSMs and SEs.

## Additional file

**Additional file 1: Table S1.** Standard *S. aureus* strains. **Table S2.** Plasmids and primers used in this study. **Table S3.** Determination of PSMs in standard *S. aureus* strains.

## Abbreviations

PSMs: staphylococcal phenol-soluble modulins
SFP: staphylococcal food poisoning
SEs: staphylococcal enterotoxins
SarA: staphylococcal accessory regulator A
Agr: accessory gene regulator
MRSA: Methicillin-resistant *S. aureus*
CA-MRSA: Community-associated MRSA
HA-MRSA: Hospital-associated MRSA
