# Supplementary material for: Expression and regulation of phenol-soluble modulins and enterotoxins in foodborne Staphylococcus aureus
Source: AMB Express. 2018 Nov 22;8:187. doi: 10.1186/s13568-018-0717-x (PMC6250609; doi:10.1186/s13568-018-0717-x)
Supplement: Supplementary file 1 — Additional file 1: Table S1. Standard S. aureus strains. Table S2. Plasmids and primers used in this study. Table S3. Determination of PSMs in standard S. aureus strains. [file 13568_2018_717_MOESM1_ESM.docx]

**Table S1** Standard *S. aureus* strains

| No. | Number & Description | Origin |
| --- | --- | --- |
| C1 | CGMCC1.0072, Huanghai1302 | China |
| C2 | CGMCC1.0089, ATCC 6538P | USA |
| C3 | CGMCC1.0128, 207-P | China |
| C4 | CGMCC1.0800, 47F2 | China |
| C5 | CGMCC1.0801, 75F2 | China |
| C6 | CGMCC1.0879 | China |
| C7 | CGMCC1.1361 | USA |
| C8 | CGMCC1.1476, Cowan I | Japan |
| C9 | CGMCC1.1477, Wood 46 | Japan |
| C10 | CGMCC1.1529 | USA |
| C11 | CGMCC1.1697 | England |
| C12 | CGMCC1.1861, ATCC 6538P | China |
| C13 | CGMCC1.2155, FDA 209P | China |
| C14 | CGMCC1.2386, ATCC 25923 | China |
| C15 | CGMCC1.2465, ATCC 6538 | USA |
| C16 | CGMCC1.2910, ATCC 6538P, JCM 2151 | Japan |
| C17 | CGMCC1.8721, ATCC29213 | USA |
| C18 | ATCC25904, Newman | USA |
| C19 | ATCC1707, USA400 | USA |
| C20 | ATCC1556, USA300 | USA |

**Table S2** Plasmids and primers used in this study

| Plasmid/primer name | Description/sequence | Reference/source |
| --- | --- | --- |
| Plasmids |  |  |
| pEC1 | pBluescript derivative, source of *ermB* gene, Ap^r^ | Zhao et al. 2010 |
| pBT2 | Shuttle vector, temp sensitive, Ap^r^ Cmr | Zhao et al. 2010 |
| pBT2-AGR | pBT2 containing 400-bp upstream and 400-bp downstream fragments of *agr* and *ermB* gene, for *agr* mutagenesis, Ap^r^ Cm^r^ Em^r^ | This study |
| pBT2-RNAIII | pBT2 containing 400-bp upstream and 400-bp downstream fragments of *RNAIII* and *ermB* gene, for *RNAIII* mutagenesis, Ap^r^ Cm^r^ Em^r^ | This study |
| pBT2-AGRA | pBT2 containing 400-bp upstream and 400-bp downstream fragments of *argA* and *ermB* gene, for *agrA* mutagenesis, Ap^r^ Cm^r^ Em^r^ | This study |
| pBT2-SARA | pBT2 containing 400-bp upstream and 400-bp downstream fragments of *sarA* and *ermB* gene, for *sarA* mutagenesis, Ap^r^ Cm^r^ Em^r^ | This study |
| pBT2-ROT | pBT2 containing 400-bp upstream and 400-bp downstream fragments of *rot*and *ermB* gene, for *rot* mutagenesis, Ap^r^ Cm^r^ Em^r^ | This study |
| Primers (5’ to 3’) |  |  |
| Up-agr-f-EcoRI | GCCgaattcATACTTAAAAATATAGTGAT | This study |
| Up-agr-r-XbalI | GCGtctagaTAACTTCATTCATTATTATA | This study |
| Down-agr/RNAIII-f-XhoI | GCGctcgagCTTTTTTGTAATGAAGAAGG | This study |
| Down-agr/RNAIII-r-SalI | GCGgtcgacTCGATAGTTATAAACCCAAA | This study |
| Up-RNAIII-f-EcoRI | GCCgaattcTAAAATGTTAAATTCGTAAT | This study |
| Up-RNAIII-r-XbalI | GCGtctagaTATTAAAACATGCTAAAAGC | This study |
| Up-agrA-f-EcoRI | GCCgaattcACAAGAAATGAATATTCCGA | This study |
| Up-agrA-r-XbalI | GCGtctaga ACATTCACATCCTTATGGCT | This study |
| Down-agrA-f-XhoI | GCGctcgagTAAGATAATAAAGTCAGTTA | This study |
| Down-agrA-r-SalI | GCGgtcgac CAACATTACAAGAGGTTGAA | This study |
| Up-sarA-f-EcoRI | GCCgaattcACTTGGAAGAGTTAAGCTAT | This study |
| Up-sarA-r-XbalI | GCGtctagaGTTTAAAACCTCCCTATTTG | This study |
| Down-sarA-f-XhoI | GCGctcgagTTTTGTTTAGCGCAATTTGG | This study |
| Down-sarA-r-SalI | GCGgtcgacTGATTAAAGATAAGAATCAT | This study |
| Up-rot-f-EcoRI | GCCgaattcTACAAATCATATCTTGAAAA | This study |
| Up-rot-r-XbalI | GCGtctagaCCCAACAATCCCGAAACTTG | This study |
| Down-rot-f-XhoI | GCGctcgagGTTTAATAGCATAAAAAGAG | This study |
| Down-rot-r-SalI | GCGgtcgacCCATCTTTAACCTAGGTTAT | This study |
| em-F- XbaI | GCGtctagaGATACAAATTCCCCGTAGGC | Shang et al. 2009 |
| em-R- XHOI | GCGctcgagGAAATAGATTTAAAAATTTCGC | Shang et al. 2009 |

**Table S3 Determination of PSMs in standard S. aureus strains**

| No. | β1 | β2 | δ | α1 | α2 | α3 | α4 |
| --- | --- | --- | --- | --- | --- | --- | --- |
| C1 | 0.228 | 0.010 | 0.003 | 0.009 | 0.022 | 0.004 | ND |
| C2 | 0.036 | 0.064 | 0.588 | 0.458 | 0.544 | 0.369 | 0.075 |
| C3 | 0.104 | 0.017 | 0.315 | 0.224 | 0.083 | 0.233 | 0.200 |
| C4 | 0.001 | ND | ND | ND | ND | ND | ND |
| C5 | ND | ND | ND | ND | ND | ND | ND |
| C6 | 0.004 | ND | 0.001 | 0.001 | ND | 0.004 | ND |
| C7 | ND | ND | ND | ND | ND | ND | ND |
| C8 | ND | ND | ND | ND | ND | ND | ND |
| C9 | ND | ND | 0.139 | 0.053 | ND | 0.004 | ND |
| C10 | 0.118 | 0.992 | 0.003 | 0.001 | ND | ND | ND |
| C11 | ND | ND | 0.007 | 0.002 | ND | ND | ND |
| C12 | 0.047 | 0.012 | 0.990 | 0.573 | 0.096 | 0.309 | ND |
| C13 | ND | ND | 0.001 | ND | ND | ND | ND |
| C14 | 0.009 | 0.017 | ND | ND | ND | ND | ND |
| C15 | 0.212 | 0.365 | 0.366 | 0.318 | 0.233 | 0.345 | ND |
| C16 | 0.510 | 3.135 | 0.080 | 0.118 | 0.148 | 0.155 | 0.017 |
| C17 | 0.564 | 0.589 | 0.272 | 0.177 | 0.106 | 0.141 | 0.048 |
| C18 | 0.006 | ND | ND | ND | ND | ND | ND |
| C19 | 0.067 | 0.089 | ND | 0.033 | 0.048 | 0.066 | ND |
| C20 | 1.000 | 1.000 | 1.000 | 1.000 | 1.000 | 1.000 | 1.000 |
